# Ovarian Vein Thrombosis Complicated by Pulmonary Embolism after Cesarean Delivery in the Presence of a Large Fibroid: Case Report and Literature Review of Contributing Factors

**DOI:** 10.1155/2021/6389713

**Published:** 2021-09-08

**Authors:** Elie S. Finianos, Stephanie F. Yacoub, Mary F. Chammas

**Affiliations:** Department of Obstetrics and Gynecology, St George University Medical Center, Beirut, Lebanon

## Abstract

Ovarian vein thrombophlebitis is rare and mostly occurs during the puerperal period and in higher rates after a cesarean delivery. The objective of this case report and literature review is to highlight the rare occurrence of an ovarian vein thrombosis in a 37-year-old woman postcesarean delivery in the setting of a large uterine fibroid who subsequently developed a pulmonary embolism. The patient presented with severe abdominal pain, fever, and chills. Imaging showed a right ovarian vein thrombosis. Following initiation of anticoagulation therapy, she developed dyspnea and testing showed a subsegmental pulmonary embolism. Further investigation showed that the patient had an undiagnosed thrombophilia thus meeting the classic Virchow triad. Complete clinical recovery was observed, and anticoagulation therapy was continued for 1 year. Our case highlights the importance of recognizing ovarian vein thrombosis because of the risk of fatal complications such as pulmonary embolus. The presence of large fibroids should raise our awareness for OVT in the setting of abdominal pain and fever. The absence of complications in previous pregnancies should not alter our clinical suspicion.

## 1. Introduction

Ovarian vein thrombophlebitis (OVT) is a rare but severe form of septic pelvic thrombophlebitis that most commonly occurs in the puerperal period, with a higher incidence after cesarean compared to vaginal delivery (1/800 vs. 1/9000) [1]. Here, we describe the case of a 37-year-old woman with a large uterine fibroid who developed a pulmonary embolism after cesarean delivery, found to have an OVT.

## 2. Case Report

A 37-year-old multiparous woman presented to the emergency department with severe right lower quadrant abdominal pain, fever and chills, one episode of vomiting, and frequent episodes of watery diarrhea. She was 9 days postpartum and had undergone an uneventful repeat cesarean section delivery following preterm rupture of membranes and preterm labor at 31 weeks of gestation. Her pregnancy had been complicated by preterm contractions and bleeding throughout due to the presence of a 13 cm uterine fibroid. Patient had been discharged home with prophylactic anticoagulation and antibiotics as per routine postoperative postpartum care. Review of systems was otherwise normal.

**Figure 1:**
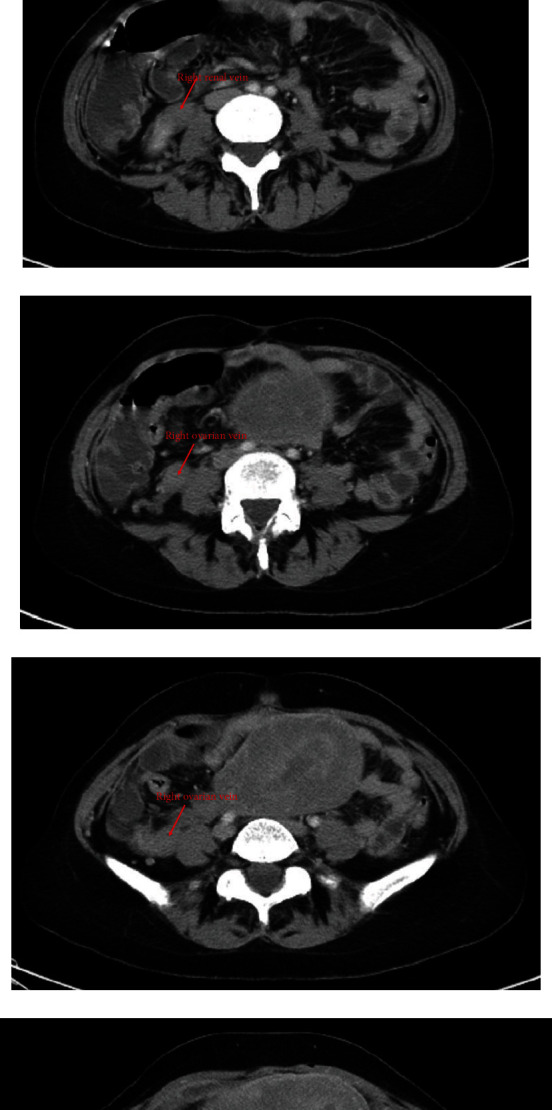
Follow-up of the (a–c) right renal vein to the (d–f) right ovarian vein. (g) No flow.

On presentation, pain was described as 8-10/10 in intensity, waxing and waning in nature, and relieved with bowel movements. Vitals in the emergency department were stable, except for a fever of 39.3°C corrected. McBurney sign was positive, and patient had signs of positive peritoneal irritation on physical exam. Apart from a CRP of 30.57, laboratory findings including a routine CBC, electrolytes, liver function tests, and urine analysis were normal. Blood, stool, and urine cultures were taken. Gastroenterology team was consulted, and a KUB X-ray was ordered (normal) and followed by a CT scan to rule out acute appendicitis. CT scan showed a normal appendix; however, a right ovarian vein thrombosis was detected. There was no associated extension into the inferior vena cava (Figure 1).

**Figure 2:**
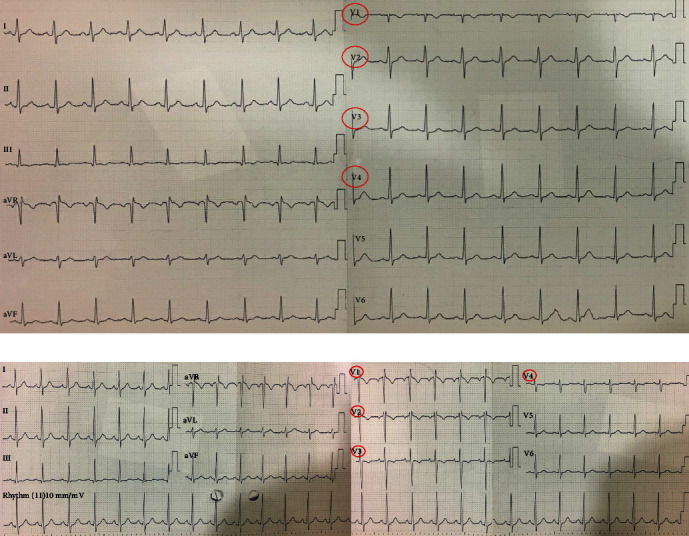
(a) EKG on admission. (b) After distress.

After consulting vascular team, the patient was started on therapeutic anticoagulation with low molecular weight heparin (LMWH) (Enoxaparin 1 mg/kg body weight subcutaneously twice daily). She was also administered Ciprofloxacin and Metronidazole for antibiotic-associated diarrhea whilst awaiting culture results. Upon transfer to the floor, patient began to experience mild dyspnea and respiratory distress, BP 120/80 mmHg, HR 110 bpm, and saturation 98% on room air. EKG showed changes in leads V1 through V4, suggestive of a new right bundle branch block (Figure 2).

Echocardiography showed right heart strain with an increase in pulmonary artery pressure. The diagnosis of a subsegmental pulmonary embolism was confirmed with CT pulmonary embolism (PE) protocol. The patient was stabilized in the intensive care unit for 48 hours and transferred to the floor stable apart from persistent diarrhea. Stool sampling showed positive *Clostridium difficile* antigen and Toxin A and so Vancomycin 125 mg PO every 6 hours was started.

Cardiovascular panel showed that the patient was homozygous for the MTHFR gene and heterozygous for Factor V Leiden. She was switched from LMWH to a Factor Xa inhibitor (Rivaroxaban 15 mg twice daily) and discharged home for a treatment of 1 year along with 5 mg of folic acid daily.

To note, 2 days prior to admission for OVT, patient presented to the emergency department 1 week postpartum with fever. CT scan at the time showed the 13 cm fibroid and normal findings postcesarean delivery and no sign of ovarian vein anomaly. She was found to have a urinary tract infection and had been sent home with PO antibiotics.

## 3. Discussion

OVT most commonly involves the right ovarian vein (70-90%) due to compression caused by the dextroverted uterus on the longer right ovarian vein at the location of the pelvic brim [2, 3]. Risk factors include infection, venous stasis, and a hypercoagulable state—all factors that are present in the puerperal setting. Other predisposing factors are congenital thrombophilias, pelvic infection, underlying malignancy, induced abortion, and the presence of large pelvic masses such as leiomyomas [3–7]. According to Shiota et al., vascular stasis occurs when the inferior vena cava is compressed by large pelvic masses that can then cause an OVT [8].

Our patient met the classic triad of Virchow that predisposes patients to thromboembolic events as well as other factors. Firstly, pregnancy is a prothrombotic event where there is an alteration in the balance between prothrombotic and anticoagulant factors increasing fibrin deposition and decreasing fibrinolysis [9]. Also, uterine infection, often encountered after cesarean delivery, causes endothelial cell damage and can increase the chances of OVT. The second risk factor was the previously undiagnosed thrombophilia. The patient had no history of miscarriages or arrested pregnancies, no embolic events, and no positive family history. A hypercoagulability workup should be considered in patients without any obvious risk factors [10]. Congenital or acquired thrombophilias are diagnosed in up to 50% of women who have a thrombotic event during pregnancy [9]. It is important to note that this patient also developed an OVT in spite of the routine prophylactic anticoagulation and antibiotics administered as part of the routine postpartum care at our hospital.

The differential diagnosis for fever and right lower quadrant pain is quite vast. It includes appendicitis, ovarian torsion, volvulus, pelvic abscesses, pyelonephritis, and endometritis [10, 11]. Due to the fact that the patient had fever, gastrointestinal symptoms, and a positive urine analysis, the diagnosis was definitely challenging. Blood cultures in patients with OVT are positive in less than 1/3 of cases [10]. Diagnostic imaging workup in these cases includes ultrasound, MRI, and CT scan with 52%, 92%, and 100% respective sensitivities in diagnosis [12–14].

Pulmonary emboli complicate less than 3% of OVT cases [15]. Emboli tend to be very small and rarely cause hypoxia, which may explain the absence of findings on the CT despite having high suspicion on clinical and indirect findings [16].

Management of these cases includes antibiotics and anticoagulation, although a general consensus or algorithm has not yet been developed. Recommendations for now include anticoagulation, mainly with LMWH and broad spectrum antibiotics with Gram negative, positive, and anaerobic coverage until clinical and laboratory improvement is seen for 48 hours [17]. Our patient developed a pulmonary embolism despite having been started on full anticoagulation, and so the decision was then made to maintain treatment for 12 months.

## 4. Conclusion

Our case highlights the importance of recognizing ovarian vein thrombosis because of the risk of fatal complications such as pulmonary embolus. The presence of large fibroids should raise our awareness for OVT in the setting of abdominal pain and fever. The absence of complications in previous pregnancies should not alter our clinical suspicion.

## Data Availability

There is no data availability statement for this case report.

## References

[1] Rottenstreich A., Da'as N., Kleinstern G., Spectre G., Amsalem H., Kalish Y. (2016). Pregnancy and non-pregnancy related ovarian vein thrombosis: clinical course and outcome. *Thrombosis Research*.

[2] Khlifi A., Kebaili S., Hammami M. (2010). Postpartum ovarian vein thrombophlebitis: report of a case and review of the literature. *North American Journal of Medical Sciences*.

[3] Dougan C., Phillips R., Harley I., Benson G., Anbazhagan A. (2016). Postpartum ovarian vein thrombosis. *The Obstetrician & Gynaecologist*.

[4] Wu C., Huang K., Liu J. (2011). Ovarian vein thrombosis associated with compression by a uterine myoma. *European Journal of Obstetrics & Gynecology and Reproductive Biology*.

[5] Cornette J., van der Wilk E., Janssen N. M. (2014). Uterine artery pseudoaneurysm requiring embolization during pregnancy. *Obstetrics and gynecology*.

[6] Fernandes F., Dinardo C., Terra-Filho (2014). Uterine myoma as a cause of iliac vein thrombosis and pulmonary embolism: common disease, rare complication. *Respirology Case Reports*.

[7] Ramanan S., Chapman-Wardy J., Watson R. (2016). Bleeding versus clotting: a complex case of a large fibroid uterus causing menorrhagia and a DVT. *Case Reports in Obstetrics and Gynecology*.

[8] Shiota M., Kotani Y., Umemoto M. (2011). Deep-vein thrombosis is associated with large uterine fibroids. *The Tohoku Journal of Experimental Medicine*.

[9] Srivastava M., Bhatnagar P., Gupta M. (2015). Deep vein thrombosis in post-partum case of caesarean section: a case report. *International Journal of Scientific Study*.

[10] Klima D., Snyder T. (2008). Postpartum ovarian vein thrombosis. *Obstetrics & Gynecology*.

[11] Salomon O., Apter S., Shaham D. (1999). Risk factors associated with postpartum ovarian vein thrombosis. *Thrombosis and Haemostasis*.

[12] Twickler D., Setiawan A., Evans R. (1997). Imaging of puerperal septic thrombophlebitis: prospective comparison of MR imaging, CT, and sonography. *American Journal of Roentgenology*.

[13] Laifer-Narin S. L., Kwak E., Kim H., Hecht E. M., Newhouse J. H. (2014). Multimodality imaging of the postpartum or posttermination uterus: evaluation using ultrasound, computed tomography, and magnetic resonance imaging. *Current Problems in Diagnostic Radiology*.

[14] Herek D., Kocyigit A., Yagci A. B. (2015). Total right ovarian vein thrombosis after cesarean section. *The Journal of Emergency Medicine*.

[15] Kominiarek M. A., Hibbard J. U. (2006). Postpartum ovarian vein thrombosis: an update. *Obstetrical & Gynecological Survey*.

[16] Oda Y., Fujita M., Motohisa C., Nakata S., Shimada M., Komatsu R. (2018). Pulmonary embolism caused by ovarian vein thrombosis during cesarean section: a case report. *JA Clinical Reports*.

[17] Al-toma A., Heggelman B. G., Kramer M. H. (2003). Postpartum ovarian vein thrombosis: report of a case and review of literature. *The Netherlands Journal of Medicine*.

